# Establishing Medical Intelligence—Leveraging Fast Healthcare Interoperability Resources to Improve Clinical Management: Retrospective Cohort and Clinical Implementation Study

**DOI:** 10.2196/55148

**Published:** 2024-10-31

**Authors:** Alexander Brehmer, Christopher Martin Sauer, Jayson Salazar Rodríguez, Kelsey Herrmann, Moon Kim, Julius Keyl, Fin Hendrik Bahnsen, Benedikt Frank, Martin Köhrmann, Tienush Rassaf, Amir-Abbas Mahabadi, Boris Hadaschik, Christopher Darr, Ken Herrmann, Susanne Tan, Jan Buer, Thorsten Brenner, Hans Christian Reinhardt, Felix Nensa, Michael Gertz, Jan Egger, Jens Kleesiek

**Affiliations:** 1 Institute for Artificial Intelligence in Medicine University Hospital Essen Essen Germany; 2 Deptartment of Hematology and Stem Cell Transplantation, West German Cancer Center, German Cancer Consortium Partner Site Essen, Center for Molecular Biotechnology University Hospital Essen Essen Germany; 3 Institute of Pathology University Hospital Essen Essen Germany; 4 Department of Neurology University Hospital Essen Essen Germany; 5 Department of Cardiology and Vascular Medicine, West German Heart- and Vascular Center University Hospital Essen Essen Germany; 6 Department of Urology and German Cancer Consortium (DKTK) Partner Site University Hospital Essen Essen Germany; 7 Department of Radiotherapy University Hospital Essen Essen Germany; 8 Department of Endocrinology, Diabetes and Metabolism University Hospital Essen Essen Germany; 9 Institute of Medical Microbiology University Hospital Essen Essen Germany; 10 Department of Anesthesiology and Intensive Care Medicine University Hospital Essen Essen Germany; 11 Institute of Computer Science Heidelberg University Heidelberg Germany

**Keywords:** clinical informatics, FHIR, real-world evidence, medical intelligence, interoperability, data exchange, clinical management, clinical decision-making, electronic health records, quality of care, quality improvement

## Abstract

**Background:**

FHIR (Fast Healthcare Interoperability Resources) has been proposed to enable health data interoperability. So far, its applicability has been demonstrated for selected research projects with limited data.

**Objective:**

This study aimed to design and implement a conceptual medical intelligence framework to leverage real-world care data for clinical decision-making.

**Methods:**

A Python package for the use of multimodal FHIR data (FHIRPACK [FHIR Python Analysis Conversion Kit]) was developed and pioneered in 5 real-world clinical use cases, that is, myocardial infarction, stroke, diabetes, sepsis, and prostate cancer. Patients were identified based on the *ICD-10* (*International Classification of Diseases, Tenth Revision*) codes, and outcomes were derived from laboratory tests, prescriptions, procedures, and diagnostic reports. Results were provided as browser-based dashboards.

**Results:**

For 2022, a total of 1,302,988 patient encounters were analyzed. (1) Myocardial infarction: in 72.7% (261/359) of cases, medication regimens fulfilled guideline recommendations. (2) Stroke: out of 1277 patients, 165 received thrombolysis and 108 thrombectomy. (3) Diabetes: in 443,866 serum glucose and 16,180 glycated hemoglobin A_1c_ measurements from 35,494 unique patients, the prevalence of dysglycemic findings was 39% (13,887/35,494). Among those with dysglycemia, diagnosis was coded in 44.2% (6138/13,887) of the patients. (4) Sepsis: In 1803 patients, *Staphylococcus epidermidis* was the primarily isolated pathogen (773/2672, 28.9%) and piperacillin and tazobactam was the primarily prescribed antibiotic (593/1593, 37.2%). (5) PC: out of 54, three patients who received radical prostatectomy were identified as cases with prostate-specific antigen persistence or biochemical recurrence.

**Conclusions:**

Leveraging FHIR data through large-scale analytics can enhance health care quality and improve patient outcomes across 5 clinical specialties. We identified (1) patients with sepsis requiring less broad antibiotic therapy, (2) patients with myocardial infarction who could benefit from statin and antiplatelet therapy, (3) patients who had a stroke with longer than recommended times to intervention, (4) patients with hyperglycemia who could benefit from specialist referral, and (5) patients with PC with early increases in cancer markers.

## Introduction

Electronic health records (EHRs) were developed in the 1970s and have evolved and grown in usage over the years [1,2]. An important milestone in the use of routinely collected clinical data was the publication of Medical Information Mart for the Intensive Care (MIMIC-III). Now in its fourth version, it was the first, large, granular publicly available patient-level dataset and includes more than 50,000 unique intensive care unit patients [3,4]. While numerous other publicly available intensive care unit databases have recently been released, the use of hospital-wide EHR data is strikingly less common [5]. More recently, implementation of EHRs has been mandated in the United States and they are the accepted standard in many other countries [6,7].

Although there have been many improvements in the EHRs, various challenges using EHR data remain. The data are large-scale (a large volume), heterogeneous (drawn from different resources), multimodal (multiple data modalities), temporal (collected over time), high-dimensional (thousands of distinct medical events), and often uncurated (not carefully chosen and thoughtfully organized or presented), poor quality (rarely subject to data quality audits), sparse (many zero values), and incomplete (missing values) [8]. Furthermore, not all patient data are located within a unified EHR. Even within a single hospital, the information is distributed across many primary systems, for example, laboratory, pathology, or medication systems—to name just a few. Within an average hospital, the number of these primary systems can easily add up to hundreds.

FHIR (Fast Healthcare Interoperability Resources) has been proposed to unify information exchange. It is a standardized set of application programming interfaces that enables the secure and efficient exchange of health care data across platforms and operating systems [9]. It is rapidly gaining traction in the health care industry and is being adopted by a growing number of health care organizations around the world. FHIR has emerged as the de facto standard for interoperability in health care, such that it is anchored within the Office of the National Coordinator for Health Information Technology 21st Century Cures Act [10].

Using FHIR to exchange medical data may provide potential benefits in many different areas, such as clinical decision support, precision medicine, mobile health apps, wearable devices, big data analytics, and using EHRs for clinical research [11]. Current reviews demonstrate that its application thus far is limited to (clinical) research with selected narrow use cases [12-14].

While FHIR holds the potential to standardize data, various challenges persist. Most frequently named is the implementation of FHIR as an application, the complexity of the FHIR standard (including its nested structure), and the representational state transfer (RESTful) approach [14]. In particular, the complexity of the data structure makes it not readily available for processing and easy access to the end user. Various solutions have been proposed, including SMART (Substitutable Medical Applications and Reusable Technologies) on FHIR, which is gaining momentum [15,16].

In this study, we evaluate the application of FHIR for a comprehensive analysis across medical disciplines to support multimodal decision management as part of routine clinical care. We propose to call this paradigm medical intelligence, akin to the expression business intelligence, which is defined as “strategies and technologies used by enterprises for the data analysis and management of business information [17,18].

We developed a standardized data pipeline using Germany’s largest FHIR database located at the University Hospital Essen. Together with department heads and (senior) physicians, we built and iteratively evaluated intuitive dashboards that provide health care workers with relevant information in a one-stop, intuitive format. As a start, we chose 5 common hospital diseases with a high disease burden, that is, myocardial infarction (MI), prostate cancer (PC), sepsis, stroke, and diabetes. To demonstrate clinical use, we iteratively tested and evaluated the dashboards with real-world data from our hospital.

## Methods

### FHIR

The resources infrastructure of FHIR forms the basic building blocks of the standard, representing health care information such as patient demographics, medication requests and administrations, or diagnostic reports. Each resource is defined in a standard way, with a consistent structure and a set of standardized elements. FHIR resources are designed to be modular, meaning that they can be combined in various ways to represent more complex information or workflows. For example, a medication order (MedicationRequest in FHIR terminology) can be linked to a patient resource to indicate who the medication is prescribed for, and a practitioner resource can be linked to indicate who prescribed the medication [19]. This modular design makes it easy to create custom workflows and exchange information granularly. While FHIR offers many benefits, implementing and using the standard can still be challenging, especially when extracting and filtering the stored data.

### FHIR Python Analysis Conversion Kit

The clinical use cases presented technical challenges regarding the identification, retrieval, clean-up, linkage, and subsequent distillation of FHIR data. To extract meaningful insights in an abstract, user-friendly, flexible, and reproducible manner we developed an open-source, interoperable Python client, and analysis toolkit for FHIR servers (FHIRPACK [FHIR Python Analysis Conversion Kit]) [20]. FHIRPACK enables clinicians and data scientists in finding, querying, and transforming FHIR resources and generating alternative representations with better processing properties such as powerful Python data analysis toolkit (pandas) data frames [21]. These can be used to carry out data analyses, generate static data visualizations, or implement dynamic dashboards that support medical processes such as continuous monitoring or complex, data-hungry research tasks such as hypothesis generation and statistical verification.

### Cohort and Covariates

**Figure 1:**
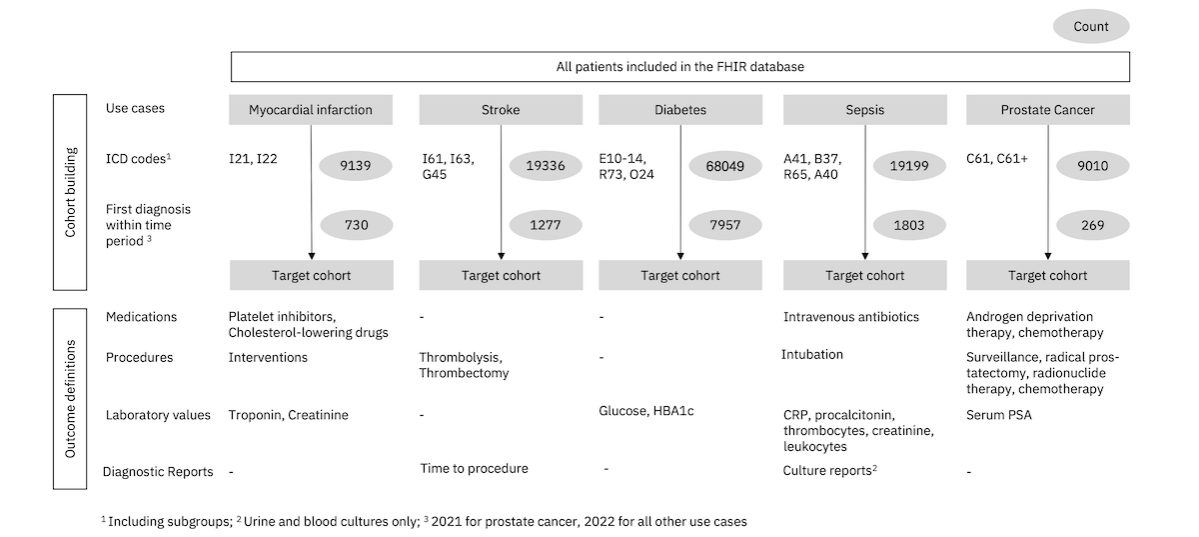
Summary of the cohort building process and outcomes studied. Gray circles indicate patient counts for each use case. CRP: C-reactive protein; FHIR: Fast Healthcare Interoperability Resources; HBA1c: glycated hemoglobin A1c; ICD: International Classification of Diseases; PSA: prostate-specific antigen.

FHIRPACK was used to access and extract relevant information from the University Hospital Essen FHIR database with over 2 billion resources. Among these are over 1.4 million unique patients—constantly growing as the EHR system is in routine use. Our extraction workflow followed a similar structure for each use case, starting with the extraction of condition resources labeled with *ICD* (*International Classification of Diseases*) codes provided by clinicians. Next, patient cohorts were built using subject references in the observational data. We then obtained specific patient information through targeted queries from their respective FHIR resource, such as laboratory values (Observation), procedures (Procedure), diagnostic reports (DiagnosticReports), drug administration (MedicationAdministration), and vital signs (Observation; Figure 1).

To enhance the speed and accuracy of our data evaluation, we restricted our cohort to a single calendar year (January 1-December 31, 2022). However, due to longer observation periods required for PC, we included all cases with diagnoses in 2021 and treatments in 2021 or 2022 for this use case. We used the FHIRPACK [20] and the Streamlit packages [22] to implement the dashboards at the point of care (PoC), accessible to clinicians in a web browser. Furthermore, we leveraged the same data and analysis processes to prepare the figures found in this manuscript using Matplotlib, another traditional Python visualization library. Details on the cohort building and covariate definitions are available in Multimedia Appendix 1.

### Ethical Considerations

This study was carried out in accordance with the Declaration of Helsinki and was approved by the Ethics Committee of the Medical Faculty of the University of Duisburg-Essen (22-10881-BO). No informed consent was required as routinely collected health care data were used.

## Results

### Cohort Description

We identified a total of 1,302,988 patient encounters in Essen University Hospital’s FHIR database for 2022 (Figure 1). Based on the *ICD-10* (*International Statistical Classification of Diseases, Tenth Revision*) codes, we identified between 269 (PC) and 7957 (diabetes) newly diagnosed patients across the 5 clinical use cases. All outcomes were derived from medication prescriptions, procedures, laboratory examinations, or diagnostic reports. While numerous data points could be reported, we focused on key indicators derived from clinicians’ inputs through iterative focus groups. The clinical rationale, research question, and implications will be discussed for each use case separately.

### MI

#### Background

An acute MI is an event of heart muscle necrosis caused by insufficient blood and oxygen supply. The incidence of acute MI or death is approximately 805,000 people in the United States alone, and accounts for approximately 20% of all causes of death [23]. Acute MI is classified based on the presence or absence of ST-segment elevation (ST elevation MI [STEMI] vs non–ST elevation MI [NSTEMI]) on electrocardiogram, while serial troponin levels differentiate a non-ST segment MI from unstable angina. Treatment includes coronary reperfusion with percutaneous coronary intervention, intravenous fibrinolytic therapy, or surgery. Depending on the bleeding risk and other patient factors, different antithrombotic drug combinations exist and are recommended in the guidelines. Further management focuses on risk reduction, including lipid-lowering drugs such as statins [24].

#### Clinical Question

How many patients were admitted with a new diagnosis of an NSTEMI or STEMI, received an interventional coronary revascularization therapy, and which antithrombotic and lipid-lowering drugs were prescribed?

#### Results

**Figure 2:**
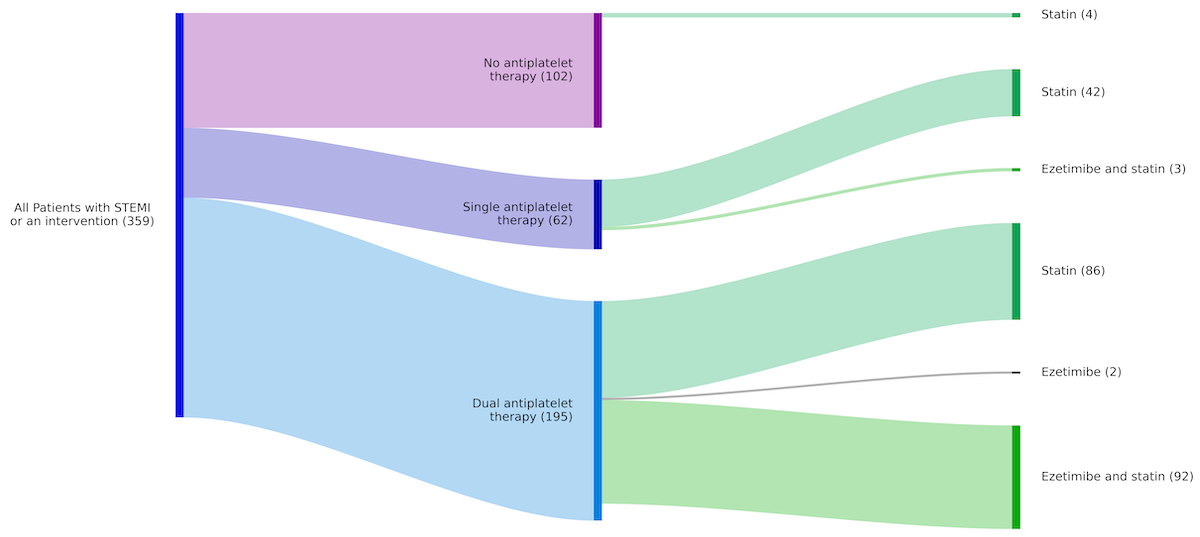
Sankey Diagram summarizing the antiplatelet (left bars) and lipid-lowering medications (right bars) prescribed to patients with STEMI or those receiving an intervention. Most patients not receiving antiplatelet therapy also did not receive lipid-lowering drugs (n=98). STEMI: ST elevation myocardial infarction.

We identified 730 patients in 2022 with a diagnosis of acute MI. Of these, 359 patients were coded as STEMI or received a coronary intervention (Figure 2). Antiplatelet therapy was started in 71.6% (257/359), with most being on dual-antiplatelet therapy, while partly single antiplatelet therapy was administered in combination with existing anticoagulation therapy (not shown). Independent of antiplatelet therapy, most patients received a statin (227/359, 63.2%), with only a minority also receiving ezetimibe (97/359, 27%). Taken together, 72.7% (261/359) of patients received guideline-recommended therapy. Meanwhile, it should be appreciated that additional patients were considered for therapy, yet not administered due to contraindications, or received bridging therapy.

#### Clinical Implication

Platelet inhibitors and statins play an important role in the prevention of MI and mortality. Implementation of a clinical dashboard highlighting patients not receiving all recommended therapies can help to initiate these cost-efficient drugs and substantially improve patient care.

### Stroke

#### Background

Ischemic stroke is a cardiovascular disease estimated to affect approximately 5.5 million patients across the globe in 2016. With approximately 116 million disease-adjusted life years lost globally, it carries a high mortality and morbidity [25]. A stroke occurs when a circulatory block results in a lack of oxygen delivery thus resulting in the subsequent death of brain tissue. In the case of an ischemic stroke, the main treatment is the resolution of the blood clot, either by drugs or mechanically by catheterization. The time from symptom onset to the resolution of the clot is one of the most important predictors of outcomes and an important indicator of improved patient care [26]. Huge efforts are made to reduce the time of the rescue chain to a minimum (“time is brain”). Along this way, the reduction of the time from presentation in the emergency room to initiation of treatment, that is, thrombolysis (“Door to Needle”) or thrombectomy (“Door to Groin”), is an important contribution of the intrahospital treatment [26]. Even though the outcome after acute ischemic stroke is generally assessed after 90 days, early neurological improvement is a surrogate indicator for a 90-day outcome. It is often defined as the percentage change in the National Institutes of Health Stroke Scale (NIHSS) score from baseline to 24 hours thereafter [27].

#### Clinical Questions

Are there fluctuations in the process quality, as measured by the waiting time between emergency room arrival and treatment application from the time of arrival in the emergency room until treatment? Does the time between the onset of symptoms until the start of a procedure correlate with early neurologic improvement?

#### Results

**Figure 3:**
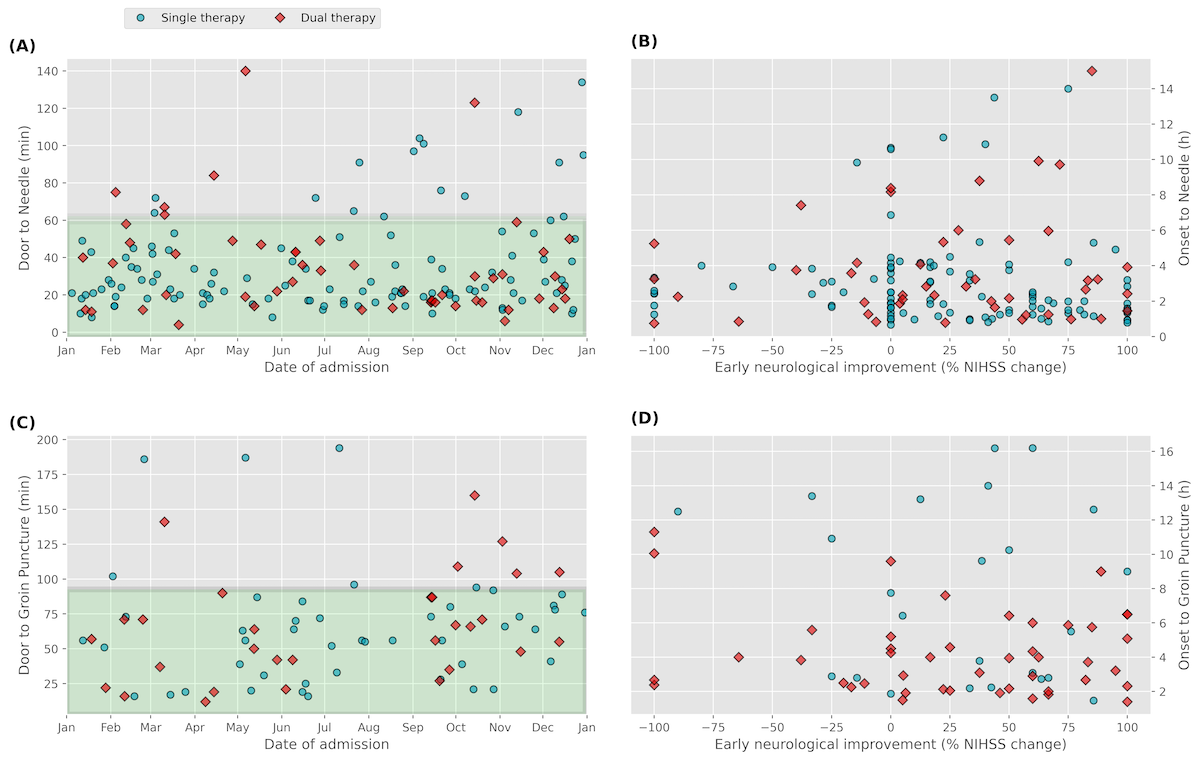
(A) Visualization of individual patients’ time from admission to the administration of thrombolysis; green area indicates the recommended time of 60 minutes and (B) correlation of early neurological improvement with time from symptom onset to needle. (C) Individual patients’ time from admission to thrombectomy; green area indicates the recommended time of 90 minutes and (D) correlation of early neurological improvement with time from symptom onset to groin puncture time. NIHSS: National Institutes of Health Stroke Scale.

In the study period, we identified 1277 patients with a stroke diagnosis treated in the department of neurology, of which 1088 were ischemic and 189 were hemorrhagic. Out of 165 patients treated with intravenous thrombolysis, 143 (86.7%) received this therapy within the guideline-recommended time window of less than 60 minutes (Figure 3). Similarly, we identified 108 patients who received thrombectomy, of which 82 (75.9%) were within the guideline-recommended time window of 90 minutes. For both therapies, our cohort of a referral stroke center has a significant number of patients presenting in an extended time window and with the need of previous coagulation testing.

Furthermore, we correlated the time from symptom onset (witnessed onset or last known-well time) to intervention with early neurological improvement (NIHSS score) from 157 patients receiving thrombolysis and 101 receiving thrombectomy (Figures 3B and 3D). Among the patients receiving thrombolysis, 63.3% (101/157) displayed a decrease in NIHSS score (ie, clinical improvement), 15.9% (25/157) showed no change, and 21.7% (31/157) exhibited an increase in NIHSS score. Among the patients who received thrombectomy, 63% (48/76) demonstrated a decrease in NIHSS score, while 7% (5/76) showed no change, and 30% (23/76) exhibited an increase in NIHSS score. In total, 25 patients were excluded from the analysis due to ongoing weaning from mechanical ventilation after thrombectomy.

#### Clinical Implications

Monitoring of clinical workflows can identify cases with longer-than-recommended turnaround times. While many reasons for delays exist, for example, need of previous coagulation assessment in patients on oral anticoagulation or need of perfusion imaging in extended-time windows, and not all may be modifiable, some can potentially be addressed, and workflows adjusted. Structured analysis of this data is the first step for improving the care of future patients.

In all figures, patients who underwent a single therapy (either thrombolysis in Figures 3A and 3B or thrombectomy in Figures 3C and 3D) are color-coded in blue, and patients who received a dual therapy (both thrombolysis and thrombectomy) in red.

### Diabetes

#### Background

Diabetes mellitus is a metabolic disorder with a high disease burden affecting 540 million people worldwide [28]. In hospitals, diabetes is one of the most common but undetected comorbidities increasing the length of stay and risk for nosocomial complications [29]. Identifying dysglycemia is a critically necessary step to introduce affected patients to dedicated in-hospital diabetes management and offer primary prevention strategies to those with prediabetes [29].

#### Clinical Question

How many inpatients are affected by dysglycemia and are cases identified appropriately?

#### Results

**Figure 4:**
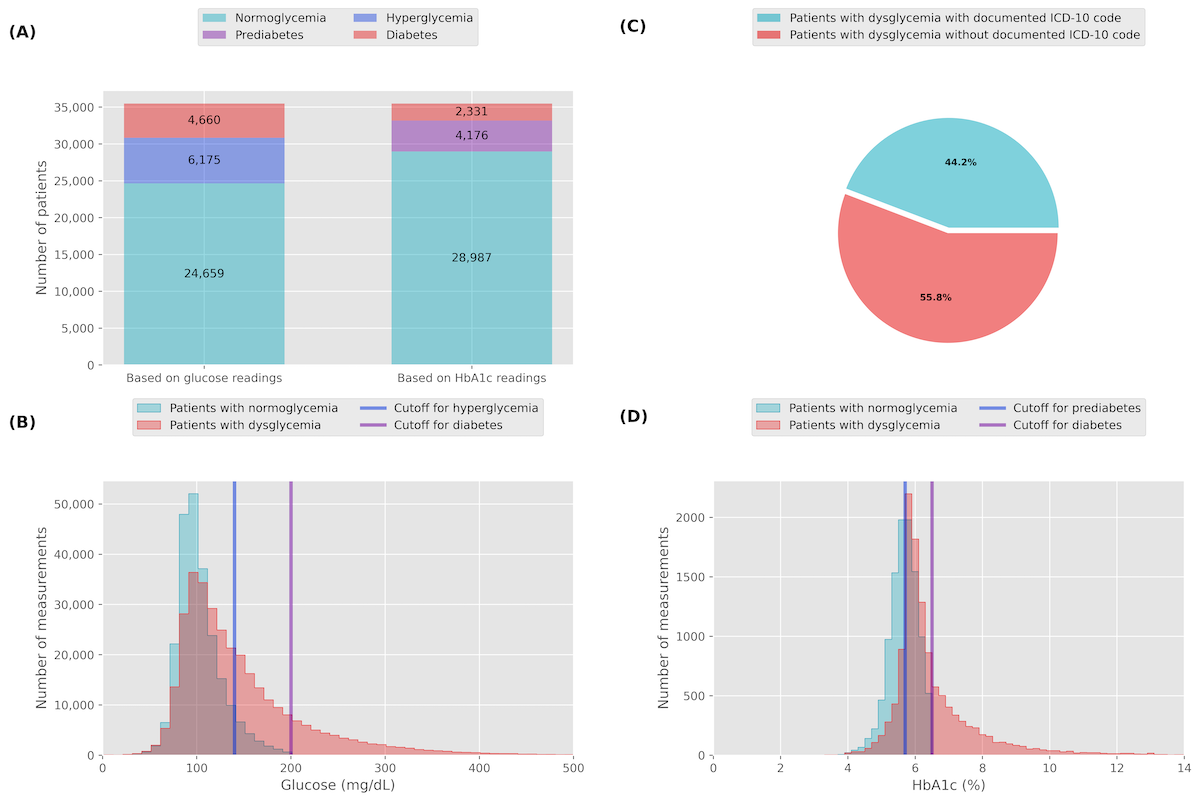
(A) Proportion of patients fulfilling the biochemical criteria for hyperglycemia (10,835/35,494, 30.5%), prediabetes (4176/35,494, 11.8%), and diabetes (5601/35,494, 15.8%), based on glucose and HbA1c readings. (C) Of all patients identified with dysglycemia (hyperglycemia, prediabetes, and diabetes), only 44.2% (6138/13,887) had a documented ICD-10 code for dysglycemia, while 55.8% (7749/13,887) of patients were not coded accordingly. Comparison of the distribution of (B) serum glucose and (D) HbA1c measurements of patients who do (blue) and do not fulfill the biochemical criteria of dysglycemia (red). Percentage of patients with biochemically proven dysglycemia with (blue; 6138/13,887, 44.2%) and without (red; 7749/13,887, 55.8%) a documented ICD-10 code. ICD-10: International Statistical Classification of Diseases, Tenth Revision.

In total, 443,866 serum glucose and 16,180 glycated hemoglobin A_1c_ (HbA_1c_) readings from 35,494 unique patients in 2022 were found. Further, 13,887 (39%) individuals, out of 35,494, were identified as having at least one pathologic glycemic finding. Overall, 30.5% (10,835/35,494) of the total cohort were at least hyperglycemic, while the prevalence of prediabetes and diabetes was 11.8% (4176/35,494) and 15.8% (5601/35,494; of which 3270 were identified by serum glucose, 941 by HbA_1c_ levels, and 1390 by both criteria), respectively (Figure 4A). Per definition, mean serum glucose and HbA_1c_ levels discriminated patients with normoglycemia from those with dysglycemia with a rise of mean glucose from 103.1 mg/dL to 112.3 mg/dL and 130.9 mg/dL to 167.1 mg/dL, respectively, and HbA_1c_ from 5.6% to 5.7% and 6% to 7.2%, respectively, in patients with normoglycemia, hyperglycemia, prediabetes, and diabetes (Figures 4B and 4D). *ICD-10* code documenting case identification for dysglycemia was found in 44.2% (6138/13,887) cases among those with biochemically proven hyperglycemia, prediabetes, or diabetes. Consequently, 55.8% (7749/13,887) of patients with dysglycemia would not have been identified by *ICD-10* coding alone (Figure 4C).

#### Clinical Implications

A dashboard visualizing in-hospital glucose metabolism data improves the dysglycemia detection rate. Future automated communication of identified cases to health care professionals may improve inpatient outcome by simplifying access by an algorithm-based referral to specialized diabetes teams as the first step of a digitalized in-hospital diabetes management program triggering dedicated diabetes care.

### Sepsis

#### Background

Sepsis is a clinically heterogeneous syndrome defined by a dysregulated host response to infection resulting in life-threatening (multi-)organ dysfunction [30]. Its primary treatment is the timely administration of broad-spectrum antibiotics. Meanwhile, with an ever-increasing prevalence of multi–drug-resistant pathogens, it is paramount to treat as targeted and shortly as possible, to avoid the selection of further resistant strains. To this end, hospital-level insights into current antibiotic use, isolated pathogens, and the prevalence of multi–drug-resistant pathogens are critical to tailoring antibiotic practice to local conditions.

#### Clinical Question

Which antibiotics are currently used in patients with sepsis? Which pathogens are isolated from urine and blood cultures and how do the inflammatory biomarkers, that is, C-reactive protein and procalcitonin, change during therapy?

#### Results

**Figure 5:**
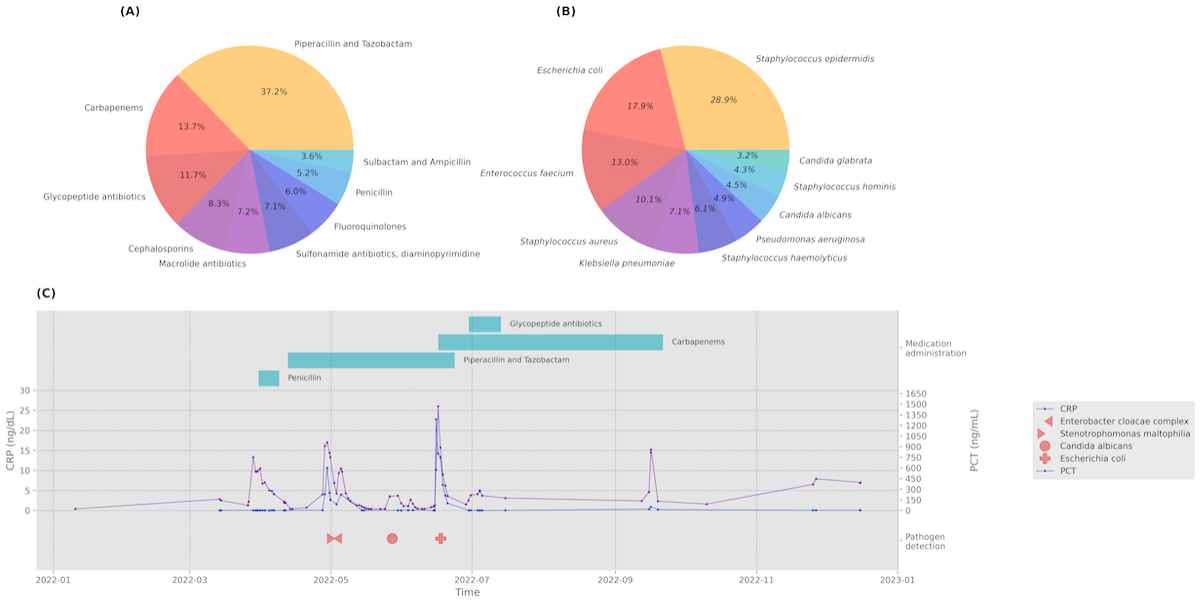
(A) Pie chart of the 10 most common antibiotics prescriptions for patients with a sepsis diagnosis. (B) Distribution of the ten most frequently isolated microorganisms. (C) Example of a patient-level graph showing changes in the inflammatory markers C-reactive protein (purple), procalcitonin (blue), isolated pathogens (red icons), and prescribed antibiotics (turquoise bars). CRP: C-reactive protein; PCT: procalcitonin.

We identified a total of 1803 patients with a sepsis diagnosis in 2022. Out of 213 distinct isolated pathogen strains, the top 10 most frequently isolated pathogen included *Staphylococcus epidermidis* (773/2672, 28.9%), a frequent contaminant with low pathogenic potential, followed by *Escherichia coli* (479/2672, 17.9%) and *Enterococcus faecium* (348/2672, 13%) strains (Figure 5A). Of the 10 most prescribed antibiotic piperacillin and tazobactam (593/1593, 37.2%), a broad-spectrum penicillin, followed by carbapenems (219/1593, 13.7%) and glycopeptides (eg, vancomycin, 186/1593, 11.7%) were most common (Figure 5B). For the clinical dashboard, we also provide a patient-level view showing the change in inflammatory markers, isolated pathogens, and antibiotics prescribed (Figure 5C).

#### Clinical Implications

The dashboard provides a summary of the evolution of inflammatory markers and antibiotic prescriptions. It can help to identify changes in the causal pathogens and monitor antibiotic prescriptions to avoid harmful over- or undertreatment.

### PC

#### Background

PC is the most common solid cancer in men in most of the Western World [31]. Various treatment options exist to achieve prolonged disease-free periods and cures. Important predictors for survival include the Tumor, Node, Metastasis stage and pathological features (International Society for Urological Pathology [ISUP] Gleason Grade Groups), which are summarized as a clinical stage (I-IV) as well as synchronous versus metachronous metastases. To assist in the diagnosis and monitor disease progression, prostate-specific antigen (PSA) is commonly used.

#### Clinical Questions

What is the current treatment landscape for PC by ISUP grades? What is the evolution of PSA levels before and after treatments depending on surgical resection status?

#### Results

**Figure 6:**
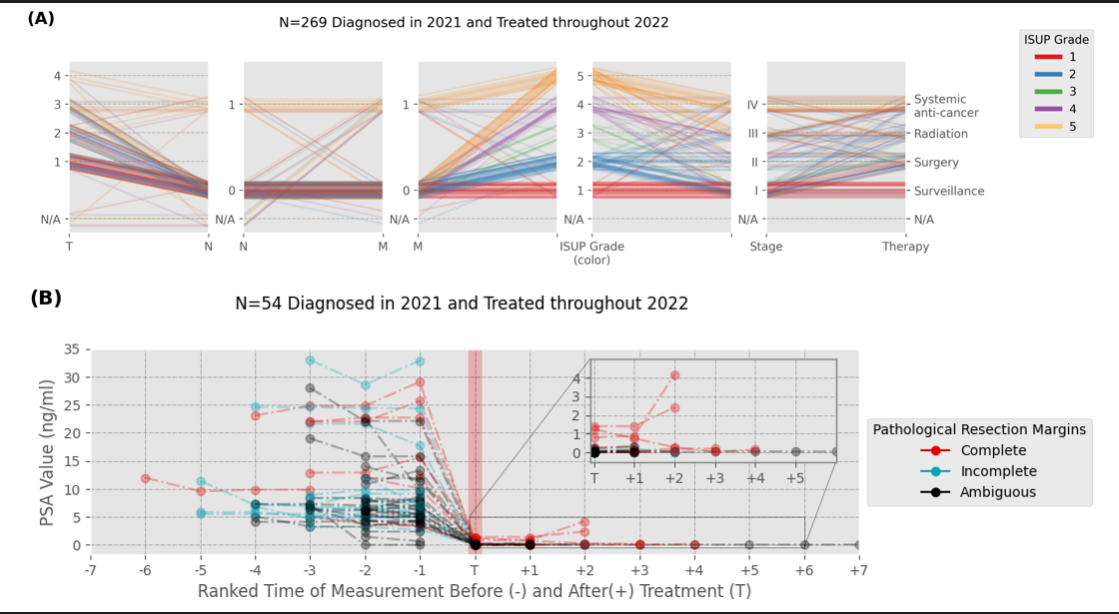
(A) Schematic summary of the Tumor, Node, Metastasis stage, International Society for Urological Pathology grade and therapies received. The color of the lines represents the International Society for Urological Pathology grades (red=1, blue=2, green=3, purple=4, and yellow=5). (B) Longitudinal evolution of prostate-specific antigen values for all (n=54) patients treated with radical prostatectomy by pathological resection margins. Normalization of the temporal axis is achieved by ranking measurements according to their corresponding timestamp, where the average time between prostate-specific antigen–measurements across patients was found to be 110 days. Zoom-in highlighting cases with rising prostate-specific antigen values after treatment, indicating biochemical persistence. PSA: prostate-specific antigen.

We identified a total of 269 patients with newly diagnosed PC in 2021 who received treatment between 2021 and 2022. Among these, localized tumors (T1N0M0) with low ISUP scores were most common at biopsy. The most common treatment approach was active surveillance (n=29, 72.5%), followed by surgery (n=8, 20%; Figure 6A). For 54 patients treated with radical prostatectomy, PSA values were monitored longitudinally, identifying 3 patients with PSA persistence or biochemical recurrence (Figure 6B).

#### Clinical Implications

Increases in PSA values after treatment indicate disease relapse or progression. Especially in the context of easy access to sensitive PSA tests, earlier detection of biochemical recurrence is warranted. Identification and monitoring can streamline treatment approaches, potentially improving patient outcomes by initiating early salvage therapy. Furthermore, with recent improvements in staging through molecular imaging with prostate-specific membrane antigen positron emission tomography or computed tomography (PSMA-PET/CT) and novel combination treatment regimens, centralized monitoring of shifts in routine practice is an essential first step to draw conclusions about the real-world effectiveness of these approaches.

## Discussion

With the introduction of EHRs, significantly more information has become available to physicians. Yet, this flood of information does not automatically result in better care, as pointedly summarized in the saying, “We are drowning in information but *starved* for knowledge” [32]. To address this, we establish a conceptual “medical intelligence” framework for leveraging real-world data for clinical monitoring and decision-making across medical specialties and at the PoC. We demonstrate the applicability of this flexible conceptual framework using 5 conditions with a high disease burden and major causes of hospitalization.

The use cases presented in this paper provide new insights for clinicians and health care managers. In the MI use case, we identified patients who did not receive lipid-lowering or antiplatelet therapy, allowing for an internal review. As for the PC use case, we detected patients with early increases in PSA values indicating tumor persistence after therapy with curative intent. We found higher than recommended use of reserve, broad-spectrum antibiotics in the sepsis use case. For patients who had a stroke, we identified some with turnaround times above guideline-recommended thresholds. Meanwhile, the diabetes use case highlighted a high prevalence of dysglycemia and diabetes in the hospital population, while coding for diabetes—and therefore potential referral rates to endocrinologists—was significantly lower.

These use cases demonstrate the applicability and benefits of a unified approach based on the interoperability of FHIR and a Python library that enables easy access to data. The approach can be quickly scaled and applied to different types of FHIR data, providing a foundation for hospitals to truly harness the power of patient data across departments, specialties, and locations. It enables easier creation of dashboards that can be used to monitor key performance indicators, summarize information for clinicians, support clinical decision-making, and ultimately facilitate better patient care. By analogy with business intelligence, where data support business decisions, our approach demonstrates that data can also support clinical decisions. We show that our approach can lead to new insights into patient care, both at the patient and hospital levels.

While the results presented are descriptive, it is possible to integrate predictive and artificial intelligence algorithms. Regardless of the use case and methodological technique, we believe that a multidisciplinary approach is key, and that successful deployment and adoption will depend on updated training for the modern physician. These “new” physicians have been called “information specialists” [33]. Information management and processing will become increasingly important with the increasing complexity and availability of data from novel diagnostic tools. Our proposed medical intelligence approach is the first step in closing the implementation gap that currently hinders clinical progress, since it provides an interoperable data structure (FHIR) with a publicly available analysis framework (this paper) [34].

The conceptual framework and implementation show that validated and highly interdisciplinary knowledge acquisition from FHIR data is possible. However, several key challenges remain. First, while FHIR provides a standardized format to exchange data, it does not dictate the content or structure of the data itself. To achieve this, FHIR profiles can be leveraged, which are structured, user-defined, and derived FHIR resources. However, these are used only infrequently or selectively and typically not hospital-wide, resulting in residual differences in how health care organizations store and structure their data. This can create challenges for interoperability and external validation of results. Second, the FHIR standard and the data it allows health care institutions to gather, and store are complex, thus requiring joint technical and medical expertise to implement. This can be a barrier to adoption for health care organizations, particularly smaller ones, that may not have the in-house expertise to develop their own organizational, conceptual, and programmatic approaches. Third, the accuracy and completeness of the data exchanged through FHIR depends on the quality of the data entered into the primary systems. To this end, incomplete or incorrect data, as is frequently the case with ICD codes, may negatively impact analytical results. Most data exist in an unstructured format that can be accessed through FHIR but requires significant postacquisition structuring.

This study provides the technical basis for pioneering medical intelligence for all institutions working with FHIR data and the results presented are directly applicable to clinical practice. However, a prospective impact on clinical decision-making remains to be demonstrated.

In summary, we developed and implemented a conceptual framework for FHIR data analysis that enables researchers and clinicians to derive insights from hospital EHR data. This approach has the potential to enable medical intelligence and improve clinical care by supporting decision-making at the PoC.

## Appendix

Multimedia Appendix 1 Additional methods.

## Abbreviations

EHR: electronic health record
FHIR: Fast Healthcare Interoperability Resources
FHIRPACK: FHIR Python Analysis Conversion Kit
HBA1c: glycated hemoglobin A1c
ICD: International Classification of Diseases
ICD-10: International Statistical Classification of Diseases, 10th Revision
ISUP: International Society for Urological Pathology
MI: myocardial infarction
MIMIC-III: Medical Information Mart for the Intensive Care
NIHSS: National Institutes of Health Stroke Scale
NSTEMI: non–ST elevation myocardial infarction
pandas: powerful Python data analysis toolkit
PC: prostate cancer
PoC: point of care
PSA: prostate-specific antigen
PSMA-PET/CT: prostate-specific membrane antigen positron emission tomography or computed tomography
SMART: Substitutable Medical Applications and Reusable Technologies
STEMI: ST elevation myocardial infarction

## Data Availability

The datasets analyzed during the current study are not publicly available as per the German legal code. However, and upon reasonable request, we will provide interested researchers with a pseudonymized version of the different datasets on an individual basis.

The FHIR data processing Python software package, FHIRPACK, is freely available on the internet, packaged, versioned, as well as citable [20]. This is the first central component to all use cases presented in this work, as it was used to retrieve, filter, process, and persist the different cohorts and their corresponding datasets. Separately, we provide the data-processing notebooks associated with each case, which produced the data and images presented herein. For reviewing purposes, the data-processing notebooks, visualizations as well as a copy of FHIRPACK’s documentation are provided [35].

## References

[1] Haux R (2006). Health information systems - past, present, future. Int J Med Inform.

[2] Saripalle R, Runyan C, Russell M (2019). Using HL7 FHIR to achieve interoperability in patient health record. J Biomed Inform.

[3] Johnson AEW, Bulgarelli L, Shen L, Gayles A, Shammout A, Horng S, Pollard TJ, Hao S, Moody B, Gow B, Lehman LH, Celi LA, Mark RG (2023). MIMIC-IV, a freely accessible electronic health record dataset. Sci Data.

[4] Moody G, Mark R G, Goldberger A L (2000). PhysioNet: a research resource for studies of complex physiologic and biomedical signals. Comput Cardiol.

[5] Sauer C, Dam TA, Celi LA, Faltys M, de la Hoz MA A, Adhikari L, Ziesemer KA, Girbes A, Thoral PJ, Elbers P (2022). Systematic review and comparison of publicly available ICU data sets-a decision guide for clinicians and data scientists. Crit Care Med.

[6] Aguirre RR, Suarez O, Fuentes M, Sanchez-Gonzalez MA (2019). Electronic health record implementation: a review of resources and tools. Cureus.

[7] Adoption of Electronic Health Records by Hospital Service Type 2019-2021.

[8] Si Y, Du J, Li Z, Jiang X, Miller T, Wang F, Jim Zheng W, Roberts K (2021). Deep representation learning of patient data from electronic health records (EHR): a systematic review. J Biomed Inform.

[9] Index - FHIR v4.3.0.

[10] (2020). Federal register. 21st Century Cures Act: Interoperability, Information Blocking, and the ONC Health IT Certification Program.

[11] Lehne M, Luijten S, Vom Felde Genannt Imbusch P, Thun S (2019). The use of FHIR in digital health - a review of the scientific literature. Stud Health Technol Inform.

[12] Vorisek CN, Lehne M, Klopfenstein SAI, Mayer PJ, Bartschke A, Haese T, Thun S (2022). Fast Healthcare Interoperability Resources (FHIR) for interoperability in health research: systematic review. JMIR Med Inform.

[13] Duda SN, Kennedy N, Conway D, Cheng AC, Nguyen V, Zayas-Cabán T, Harris PA (2022). HL7 FHIR-based tools and initiatives to support clinical research: a scoping review. J Am Med Inform Assoc.

[14] Ayaz M, Pasha MF, Alzahrani MY, Budiarto R, Stiawan D (2021). The Fast Health Interoperability Resources (FHIR) standard: systematic literature review of implementations, applications, challenges and opportunities. JMIR Med Inform.

[15] Mandl KD, Kohane IS (2009). No small change for the health information economy. N Engl J Med.

[16] SMART Health IT.

[17] Olszak CM, Ziemba E (2006). Business intelligence systems in the holistic infrastructure development supporting decision making in organisations. Interdiscip J Inf Knowl Manag.

[18] (2023). Business intelligence.

[19] Mandel J, Kreda DA, Mandl KD, Kohane IS, Ramoni RB (2016). SMART on FHIR: a standards-based, interoperable apps platform for electronic health records. J Am Med Inform Assoc.

[20] Salazar J (2022). FHIRPACK (FHIR Python Analysis Client and Kit).

[21] Reback J, McKinney W, Van Den Bossche J, Augspurger T, Cloud P, Hawkins S, Klein A, Roeschke M, Tratner J, Petersen T, She C, Ayd W, Garcia M, Schendel J, Hayden A, Saxton D, Jbrockmendel, Gfyoung, Sinhrks, MomIsBestFriend (2020). pandas-dev/pandas: Pandas 1.1.3.

[22] (2021). Streamlit: A faster way to build and share data apps.

[23] (2022). Heart disease and stroke statistics—2022 update: a report from the American Heart Association. Circulation.

[24] Collet JP, Thiele H, Barbato E, Barthélémy O, Bauersachs J, Bhatt DL, Dendale P, Dorobantu M, Edvardsen T, Folliguet T, Gale CP, Gilard M, Jobs A, Jüni P, Lambrinou E, Lewis BS, Mehilli J, Meliga E, Merkely B, Mueller C, Roffi M, Rutten FH, Sibbing D, Siontis GC M, ESC Scientific Document Group (2021). 2020 ESC guidelines for the management of acute coronary syndromes in patients presenting without persistent ST-segment elevation. Eur Heart J.

[25] GBD 2016 Stroke Collaborators (2019). Global, regional, and national burden of stroke, 1990-2016: a systematic analysis for the global burden of disease study 2016. Lancet Neurol.

[26] Campbell BCV, Khatri P (2020). Stroke. Lancet.

[27] Kobeissi H, Ghozy S, Bilgin C, Kadirvel R, Kallmes DF (2023). Early neurological improvement as a predictor of outcomes after endovascular thrombectomy for stroke: a systematic review and meta-analysis. J Neurointerv Surg.

[28] GBD 2019 Diabetes in the Americas Collaborators (2022). Burden of diabetes and hyperglycaemia in adults in the Americas, 1990-2019: a systematic analysis for the Global Burden of Disease Study 2019. Lancet Diabetes Endocrinol.

[29] Valent F, Tonutti L, Grimaldi F (2017). Does diabetes mellitus comorbidity affect in-hospital mortality and length of stay? Analysis of administrative data in an Italian academic hospital. Acta Diabetol.

[30] Singer M, Deutschman CS, Seymour CW, Shankar-Hari M, Annane D, Bauer M, Bellomo R, Bernard GR, Chiche J, Coopersmith CM, Hotchkiss RS, Levy MM, Marshall JC, Martin GS, Opal SM, Rubenfeld GD, van der Poll T, Vincent J, Angus DC (2016). The third international consensus definitions for sepsis and septic shock (Sepsis-3). JAMA.

[31] Siegel RL, Miller KD, Fuchs HE, Jemal A (2022). Cancer statistics, 2022. CA Cancer J Clin.

[32] Naisbitt J (1982). Megatrends: Ten New Directions Transforming Our Lives.

[33] Jha S, Topol EJ (2016). Adapting to artificial intelligence: radiologists and pathologists as information specialists. JAMA.

[34] Weiss CH, Krishnan JA, Au DH, Bender BG, Carson SS, Cattamanchi A, Cloutier MM, Cooke CR, Erickson K, George M, Gerald JK, Gerald LB, Goss CH, Gould MK, Hyzy R, Kahn JM, Mittman BS, Mosesón EM, Mularski RA, Parthasarathy S, Patel SR, Rand CS, Redeker NS, Reiss TF, Riekert KA, Rubenfeld GD, Tate JA, Wilson KC, Thomson CC, ATS Ad Hoc Committee on Implementation Science (2016). An official American Thoracic Society research statement: implementation science in pulmonary, critical care, and sleep medicine. Am J Respir Crit Care Med.

[35] Leveraging FHIR to Improve Clinical Management. Establishing Medical Intelligence.

