# Supplementary material for: Establishing Medical Intelligence—Leveraging Fast Healthcare Interoperability Resources to Improve Clinical Management: Retrospective Cohort and Clinical Implementation Study
Source: J Med Internet Res. 2024 Oct 31;26:e55148. doi: 10.2196/55148 (PMC11565078; doi:10.2196/55148)
Supplement: Multimedia Appendix 1 [file jmir_v26i1e55148_app1.docx]

Supplementary methods

**Use case specific cohort building and co-variate definitions**

For each clinical question hypotheses were formulated and iteratively refined both conceptually and data-wise. However, the approach, data sources, as well as data elements processed, vary greatly depending on the clinical question and research objectives. Therefore, we provide additional details on the specific cohort definition and co-variates for each use case below.

Suppl. fig. 1: Implicit data-processing workflow emerging from the inter-disciplinary and iterative approach towards answering a clinical question with the help of Extraction Transformation and Load tools, techniques, and procedures.


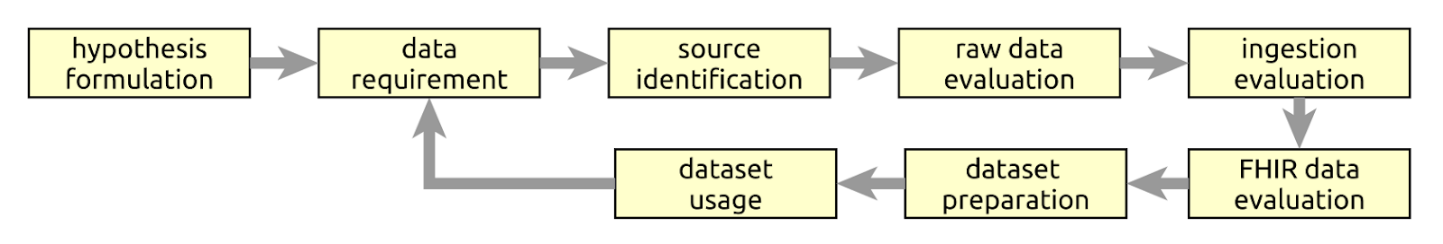


*Myocardial Infarction:*

We established a cohort using ICD-10 codes I21 and I22 to identify patients with acute myocardial infarction. To identify individuals who underwent interventions, we extracted FHIR procedures with the OPS code 8-83. The administration of anti-platelet therapy was based on the medication administration records (MedicationAdministration), including phenprocoumon, dabigatran, rivaroxaban, apixaban, edoxaban, clopidogrel, ticagrelor, prasugrel, and aspirin and the respective ATC-codes: B01AA04, B01AE07, B01AF01, B01AF02, B01AF03, B01AC04, B01AC24, B01AC22, B01AC06. As for lipid-lowering drugs we extracted data on ezetimibe as well as statin therapy, i.e., fluvastatin, atorvastatin, lovastatin, pravastatin, rosuvastatin, and simvastatin, using the ATC-codes C10AX09, C10AA05, C10AA04, C10AA02, C10AA03, C10AA07, C10AA01

*Prostate Cancer:*

Patients diagnosed with prostate cancer in 2021 and treated throughout 2022 were identified using FHIR Conditions with ICD-10 codes C61 and C61+ (Observation). The corresponding hospital internal tumor-IDs were gathered from the same resource and used to de-duplicate patient identities. The resulting mapping was used for data cleaning and proper attribution of other relevant FHIR resources (MedicationAdministration and CarePlan). Prostate Specific Antigen (PSA) and testosterone measurements, as well as clinical and pathological staging grades, were extracted from Observations and showed different levels of granularity and consistency. PSA values, clinical oncological staging, and International Society of Urological Pathology (ISUP) grades were the most populated, while testosterone data was considerably sparse and therefore excluded. Therapeutic measures were extracted from CarePlans, a by-product of internal oncological documentation processes, and the Procedure resources using the German operation and procedure classification system (OPS) codes 5-604.

*Sepsis:*

Sepsis cases were identified based on ICD-10 codes A41, B37, R65, and A40. Covariates included laboratory values obtained from FHIR Observations, i.e., C-reactive protein (CRP) and procalcitonin (PCT), as well as antibiotic administrations (MedicationAdministrations). We considered all commonly prescribed antibiotic classes available in the EU, including penicillins, e.g., flucloxacillin, penicillins with increased activity, e.g., amoxicillin-clavulanate, cephalosporins, e.g., ceftriaxone, fluoroquinolones, e.g., ciprofloxacin, lincosamides, e.g., clindamycin, macrolides, e.g., clarithromycin, tetracyclines, e.g., doxycycline, trimethoprim-sulfamethoxazole, oxazolidinones, e.g. linezolid, glycopeptides, e.g., vancomycin, carbapenems, e.g., meropenem, rifamycins, e.g. rifampicin, and aminoglycosides, e.g., gentamicin. Antibiotics were extracted from MedicationAdministrations using ATC-codes J01AA, J01CA01, J01CF05, J01CR01, J01CR02, J01CR04, J01CR05, J01CR25, J01DC02, J01DC04, J01DD02, J01DD04, J01DF01, J01DH02, J01DH51, J01EE01, J01FA01, J01FA09, J01FA10, J01GB01, J01GB03, J01GB06, J01MA02, J01MA12, J01MA14, J01XA01, J01XB01, J01XX01. Furthermore, we extracted the type of isolated pathogen from blood and urine culture reports.

*Stroke:*

Patients were identified using the stroke ICD-10 codes I61, I63 and G45 from Observation. Leveraging those, we gathered their corresponding digitalized neurological clinical documentation referenced in FHIR DiagnosticReports (PDFs) and extracted the arrival date, onset time of symptoms, thrombolysis and thrombectomy starting time, as well as the NIHSS scores at arrival and after 24 hours. Non-FHIR-compliant data such as PDFs were extracted with FHIRPACK but further processed using basic Natural language Processing (NLP) methods using the library PDFPlumber. We defined two key factors: "door-to-needle" time, the interval from arrival in the emergency room (ED) to intravenous thrombolytic therapy, and "door-to-groin" time, the duration from ED arrival to the start of endovascular treatment. To assess neurological deficits, we utilized the standardized NIH Stroke Scale (NIHSS). We measured early improvement by calculating the NIHSS change in percent: (Admission NIHSS - 24-hour NIHSS) × 100 / Admission NIHSS, which reflects neurological improvement within the first 24 hours of admission.

*Diabetes:*

Inpatients with at least one glucose and/or HbA1c FHIR Observation and/or pre-/diabetes-specific ICD-10 codes (R73.0, E10, E11, E12, E13, E14 and O24) in 2022 were gathered. Glycemic status was defined biochemically according to current guidelines:^37^
(A) HbA1c ≥6.5% (48 mmol/mol) and/or any repeated serum glucose value ≥200 mg/dl (11.1 mmol/l) as diabetes

(B) HbA1c of 5.7-6.4% (39-47 mmol/mol) as prediabetes.

(C) Glucose levels above 140 mg/dl (7.8 mmol/l) as hyperglycemia.

The presence of documented pre-/diabetes-specific ICD-10 codes was regarded as evidence that patients were appropriately identified as patients with dysglycemia.
